# Patterns of *mt*DNA variation reveal complex evolutionary history of relict and endangered peat bog pine (*Pinus uliginosa*)

**DOI:** 10.1093/aobpla/plz015

**Published:** 2019-03-14

**Authors:** Bartosz Łabiszak, Julia Zaborowska, Witold Wachowiak

**Affiliations:** 1 Institute of Environmental Biology, Adam Mickiewicz University, Poznań, Poland; 2 Institute of Dendrology, Polish Academy of Sciences, Kórnik, Poland

**Keywords:** Endangered species, genetic structure, molecular markers, phylogeography, pines

## Abstract

Estimates of genetic differentiation at intra- and interspecific level are often hindered by the lack of suitable molecular markers. Low phylogeographic resolution limits development of appropriate conservation strategies especially in case of endangered forest tree species with small and disjunct distribution. In this study, we assessed fine-scale genetic structure of relict and endangered peat bog pine (*Pinus uliginosa*) and two other closely related European pine species (*Pinus mugo* and *Pinus uncinata*) using a set of 15 newly developed maternally inherited and seed-mediated mitochondrial DNA (*mt*DNA) markers and two previously known polymorphic *mt*DNA regions (*nad1*, *nad7*). Three main groups, corresponding in general to three investigated species were revealed in the haplotype network analysis. However, only *P. uncinata* was clearly distinct at all levels of analysis, whereas great genetic similarity and haplotype sharing was observed between *P. uliginosa* and *P. mugo*. Strong phylogeographic structure was found in *P. uliginosa* that showed high differentiation at relatively short geographical distance among populations and the existence of mitochondrial lineages of different evolutionary history. Hybridization with other pine species has likely contributed to genetic differentiation of *P. uliginosa* as indicated by contemporary distribution of *mt*DNA haplotypes. The research emphasizes the importance of accurate assessments of genetic structure of endangered species with complex evolutionary history for development of efficient conservation strategies.

## Teaser

Using newly developed mitochondrial DNA markers we looked at the relationship between three closely related European pine species. We were especially interested in assessing the genetic structure of *Pinus uliginosa*—a peat bog pine uniquely adapted to the nutrient-sparse bog environment that nowadays is highly endangered due to environmental changes and habitat loss. Surprisingly, we found evidence of strong differentiation between the neighbouring populations of this species and also signatures of hybridization events, shaping its contemporary gene pool. Our results improve current taxonomic knowledge of the studied *Pinus mugo* complex and could also serve as a basis for the development of successful conservation strategies for peat bog pine.

## Introduction

Assessments of eco-evolutionary mechanisms that shape genetic structure of populations are of key importance to understand the influence of past and ongoing environmental changes on plant ecosystems. In recent years, molecular markers greatly improved our ability to assess genetic differentiation at within and among species level. However, due to genome complexity and limited access to suitable genomic resources, phylogenetic investigations remain still challenging especially in many non-model plant species (Petit and Vendramin 2007; Roy *et al.* 2010; Whitlock *et al.* 2010). Assessments of species boundaries and their underlying population structure are needed not only to improve taxonomic knowledge, but also to properly guide decision-making in conservation of endangered tree species (Newton *et al.* 1999).

European hard pine taxa contain several species intensively studied due to their ecological and social value including representatives of the *Pinus mugo* complex (Wang *et al.* 1999; Gernandt *et al.* 2005; Eckert and Hall 2006). It contains closely related taxa, some undergoing severe population decline and being hard to delimit in an unambiguous way due to low resolution of available biometric and molecular markers (Christensen 1987; Hamernik and Musil 2007). One of the most intriguing representatives of the complex is the peat bog pine (*Pinus uliginosa*). It is a single-stemmed tree up to 20 m in height, inhabiting humid and nutrient-sparse bog environments in lowlands. Originally it has been described from two sites in Central Sudetes, Poland (Neumann 1837; Wimmer 1837), and at present only a few isolated stands are known in Poland, Germany and Ukraine (Boratyński 1994). The species strict ecological specialization together with restricted, island-like range poses a high extinction risk, especially in face of warmer and drier climate that severely affects peatland plant communities (Holt 1990; Weltzin *et al.* 2003; Peterson *et al*. 2005; Audrey *et al.* 2009). In Poland, where the majority of peat bog pine populations are located, rapid decline of trees was observed in recent years. Consequently, in some populations no more than 100 specimens of peat bog pine have been left (Danielewicz and Zieliński 2000) and this taxon is considered as highly endangered and protected, at least on national scale (Polish Plants Red Book).

Interestingly, almost 100 years after it was first described, taxonomic position of this species is not fully resolved. Research to date has focused mostly on peat bog pine evolutionary history and processes shaping its genetic structure, especially in the context of the species protection. Nonetheless, these studies were mainly based on morphological features of needles and cones (Boratynska and Boratynski 2007; Boratynska and Lewandowska 2009) and on isoenzymes (Siedlewska and Prus-Głowacki 1995; Prus-Glowacki *et al.* 1998; Wachowiak and Prus-Glowacki 2009), and they were often restricted to single population and/or individuals. Studies based on morphological data place peat bog pine together with other closely related pine species from the *P. mugo* complex including dwarf mountain pine (*P. mugo*) from mountain regions of Central and Western Europe and mountain pine (*Pinus uncinata*) from Iberian Peninsula (Christensen 1987; Hamernik and Musil 2007). However, the taxa exhibit also some similarity at biometric and biochemical traits to *Pinus sylvestris* (Boratynska and Boratynski 2007) and close relationship between these taxa is reflected in phylogeny of the genus (Grotkopp *et al.* 2004; Gernandt *et al.* 2005). Shared characteristics at some traits led the authors to hypothesis that *P. uliginosa* might be a marginal population of *P. uncinata* (Krzakowa *et al.* 1984) or possibly ancient, stabilized hybrid between *P. mugo* and *P. sylvestris* (Lewandowski *et al.* 2000; Boratynska and Boratynski 2007). Some indication of relatively recent divergence of peat bog pine from other taxa from the *P. mugo* complex was found at sequence variation at nuclear genes (Wachowiak *et al.* 2011); however, the exact genetic relationship between the taxa is not conclusive.

To date, efforts to describe a range-wide phylogeographic structure for peat bog pine were limited (Heuertz *et al.* 2010; Dzialuk *et al.* 2017). This may be in part attributed to insufficient number and low resolution of molecular markers developed for the pine complex. In case of forest tree species, cytoplasmic DNA markers that are haploid and transmitted uniparentally through pollen or seeds are of particular interest for population history studies. In wind-pollinated species such as pines, mitochondrial DNA (*mt*DNA) markers, maternally inherited and dispersed by seeds on short distances, are especially valuable as they best reflect past demographic changes and longer retain patterns of demographic structure (Toth *et al.* 2017). Although *mt*DNA variation was commonly used in previous population history studies in forest tree species, the obtained resolution was very weak due to low number of available markers described for European pines (Soranzo *et al.* 2000; Cheddadi *et al.* 2006; Naydenov *et al.* 2007). Difficulties in finding new *mt*DNA markers result mostly from large size of plant mitochondria, their complex structure with numerous repeated regions and generally low rate of sequence evolution (Guo *et al.* 2016; Smith 2016). However, recent advances in sequencing technologies allowed development of novel genomic resources in non-model plant, including descriptions of a large fragment of mitochondrial genome in pines (Donnelly *et al.* 2017). Based on the polymorphisms found in the regions we developed a large set of new *mt*DNA markers that proved to be useful in population genetic studies of closely related pine species.

Here, we present the results of first large-scale study on genetic structure of relict and endangered peat bog pine with the application of newly developed *mt*DNA markers. Using a set of peat bog pine populations and a collection of a reference samples of closely related taxa we: (i) looked at the population structure of the remaining stands of the peat bog pine, (ii) assessed levels of *mt*DNA variation in *P. uliginosa* populations to infer past population history processes, (iii) examined genetic relationship of *P. uliginosa* as compared to other pine species in reference to earlier hypothesis. Based on our findings we suggest potential conservation strategies for preservation of genetic resources of the endangered peat bog pine.

## Materials and Methods

### Sampling and marker development

**Figure 1.**
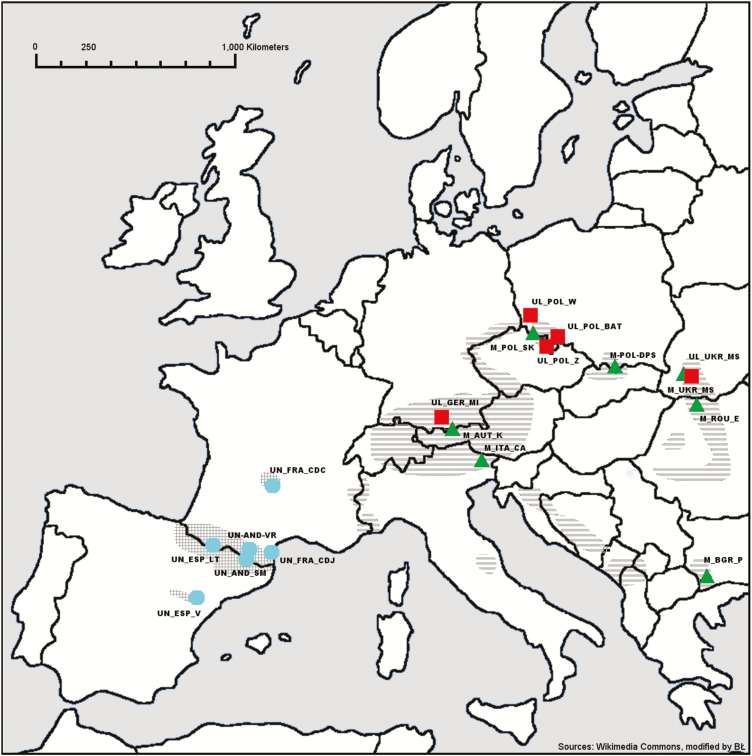
Geographic location of studied *Pinus uliginosa* populations (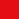) and reference stands of closely related pine species: *P. mugo* (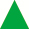) and *P. uncinata* (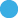). Distribution range of *P. mugo* and *P. uncinata* is marked with grey horizontal and crossed stripes, respectively. Population acronyms and exact location as in Table 1.

Five populations of *P. uliginosa* were sampled together with 13 reference populations including 7 *P. mugo* and 6 *P. uncinata* stands sampled across the European ranges of the taxa. There are no other pines closely related to the studied taxa that occur in the sympatry of the analysed populations. Sample size ranged from 8 to 40 trees per population, resulting in a total of 384 individuals analysed (Fig. 1; Table 1). Genomic DNA was extracted from needle tissues using DNeasy Plant Mini Kit (Qiagen), following standard manufacturer protocol. In order to assess genetic structure and relationships between investigated taxa we developed a large-scale, cost-effective genotyping method of individuals at multiple loci using polymorphic *mt*DNA regions described in Donnelly *et al.* (2017). Initially, a set of approximately 30 regions were screened in Nebcutter V.2.0 (Vincze *et al.* 2003) in order to find suitable Single Nucleotide Polymorphism (SNPs) for Polymerase Chain Reaction – Restriction Fragment Length Polymorphism (PCR-RFLP) analysis. PCR amplification of 15 polymorphic regions was carried out in a total volume of 15 µL containing 15 ng of template DNA, 10 µM of each dNTP, 0.2 µM each of forward and reverse primers, 0.15 U *Taq* DNA polymerase, 1× BSA, 1.5 µM of MgCl_2_ and 1× PCR buffer (Novazym). Standard amplification procedures were used with initial denaturation at 94 °C for 3 min followed by 35 cycles with 30 s denaturation at 94 °C, 30 s annealing at 60 °C for most loci and 1 min 30 s extension at 72 °C, and a final 5 min extension at 72 °C. The genotyping was done in all but one case using respective restriction enzyme and electrophoresis of restriction products in 2 % agarose gel. List of all PCR primer pairs and restriction enzymes used in this study is given in Supporting Information—Table S1. Insertion/deletion (indel) polymorphism in PR34 region was genotyped using Sanger sequencing. The respective fragments were amplified as described above and PCR fragments were purified using Exonuclease I-Shrimp Alkaline Phosphatase enzymatic treatment. About 20 ng of PCR product was used as template in 10 μL sequencing reaction with the Big Dye Terminator DNA Sequencing Kit (Applied Biosystems). CodonCode Aligner (CodonCode Corporation) was used to edit and align sequences. Additionally, two previous *mt*DNA markers including *nad7* and *nad1* were genotyped according to methods described in Jaramillo-Correa *et al.* (2004) and Soranzo *et al.* (2000), respectively.

**Table 1.** Location, sample size, corresponding SAMOVA group and basic haplotype statistics of studied pine populations. *N*—number of individuals/number of individuals genotyped; *H*_n_—number of haplotypes; *H*_d_—haplotype diversity; *H*_s_—number of singleton haplotypes.

Species/acronym	Population	Latitude N	Longitude E	Altitude (m)	*N*	*H*_n_	*H*_d_	*H*_s_	SAMOVA group
*P. uliginosa*									
UL_POL_W	Poland, Sudety Mts., Low Silesian Pinewood, Węgliniec reserve	51°17′50″	15°14′20″	190	40/40	13	0.83	7	I
UL_GER_MI	Germany, Bavaria, Mittenwald	47°28′50″	11°16′27″	856	25/21	10	0.91	4	I
UL_POL_BAT	Poland, Sudety Mts., Batorów reserve	50°27′32″	16°23′01″	710	36/33	2	0.17	0	III
UL_POL_Z	Poland, Sudety Mts., Zieleniec reserve	50°20′54″	16°24′42″	755	30/27	18	0.96	13	III
UL_UKR_MS	Ukraine, Gorgany Mts., Mshana	48°40′33″	23°55′19″	830	12/12	4	0.74	1	IV
	All				143/133	40	0.91	22	–
*P. mugo*									
M_POL_SK	Poland, Sudety Mts., Śląskie Kamienie	50°46′35″	15°36′08″	1300	10/7	2	0.48	0	I
M_POL_DPS	Poland, Tatra Mts., Dolina Pięciu Stawów	49°13′09″	20°03′05″	1700	12/12	5	0.73	3	I
M_AUT_K	Austria, Karwendel Mts., Scharnitz	47°22′42″	11°17′45″	1400	22/22	5	0.71	2	I
M_UKR_MS	Ukraine, Gorgany Mts., Mshana	48°40′33″	23°55′19″	830	8/8	1	0.00	0	IV
M_ROU_E	Romania, Eastern Carpathians, Munti Rodnei	47°34′03″	24°48′00″	1720	22/19	4	0.30	3	I
M_BGR_P	Bulgria, Pirin Mts., Vikhren	41°46′07″	23°25′22″	2000	22/22	2	0.48	0	I
M_ITA_CA	Italy, Carnic Alps, Passo di Pramollo	46°32’45″	13°15′35″	1530	21/21	3	0.19	2	I
	All				117/111	16	0.87	7	–
*P. uncinata*									
UN_AND_VR	Andorra, Eastern Pyrenees, Vall de Ransol	42°35′02″	01°38′21″	2025	22/22	1	0.00	0	II
UN_AND_SM	Andorra, Eastern Pyrenees, San Miguel de Engolasters	42°31′28″	01°34′12″	2000	22/20	3	0.42	1	II
UN_ESP_LT	Spain, Western Pyrenees, La Trapa	42°41′19″	-00°32′12″	1720	22/22	2	0.37	0	II
UN_ESP_V	Spain, Sierra de Gudar, Valldelinares	40°28′49″	-00°41′51″	2000	22/20	2	0.42	0	II
UN_FRA_CDJ	France, Eastern Pyrenees, Col de Jau	42°39′19″	02°15′22″	1520	12/12	2	0.17	1	II
UN_FRA_CDC	France, Massif Central, Col de la Croix-Morand	45°36′00″	02°50′59″	1400	24/23	4	0.58	0	II
	All				124/119	5	0.53	1	–

### *mt*DNA haplotype analysis

Multilocus genotypes were assessed for each individual using all 17 markers. All except one marker (PR29) were found to be polymorphic in investigated species and thus 16 markers were used thereafter. Individuals with level of missing data ≥ 10 % were excluded from further analysis. Phylogenetically informative gaps (indels) in PR34, *nad1* and *nad7* were coded as single mutation events for analyses. The number of haplotypes (*H*_n_) and haplotype diversity (*H*_d_) were computed at species and population level using DnaSP v.5 (Librado and Rozas 2009). A median-joining network, illustrating phylogenetic relationship among *mt*DNA haplotypes, was constructed for all sequences with PopART (Bandelt *et al.* 1999). The geographic distribution of markers was assessed at the most frequent *mt*DNA haplotypes detected (i.e. those with frequency ≥ 1 %).

### Population structure and differentiation

To show genetic relationships between populations and species, genetic distance based on all polymorphic *mt*DNA sites was calculated in MEGA 7 (Kumar *et al.* 2016) and used in principal coordinate analysis (PCoA) in GenAlEx 6.501 software (Peakall and Smouse 2006; Peakall and Smouse 2012). The genetic relationships between samples were also investigated using the unweighted pair group method with arithmetic mean (UPGMA) in MEGA 7.

The hierarchical analysis of spatial molecular variance in populations was conducted using SAMOVA 2.0 program (Dupanloup *et al.* 2002) in order to find *K* groups of maximally differentiated but geographically homogenous populations. The analysis was performed at *K* values ranging from 2 to 17. Genetic differentiation among groups identified by SAMOVA 2.0 was estimated using an analysis of molecular variance (AMOVA) implemented in Arlequin v.3.5.22 (Excoffier and Lischer 2010).

Additional measures of population differentiation (*G*_ST_, *N*_ST_) were calculated and compared to each other using a permutation test with 10 000 replicates in PermutCpSSR v.1.2.1 software (Pons and Petit 1996; Burban *et al.* 1999). The comparison between those estimates can elucidate presence of a formal phylogeographic structure in cases where *N*_ST_ value is higher than the *G*_ST_ value. Finally, isolation by distance hypothesis was verified by Mantel test using GenAlEx 6.501 software with 1000 random permutations of the relationship between genetic (based on *N*_ST_) and geographic distance matrices.

## Results

**Figure 2.**
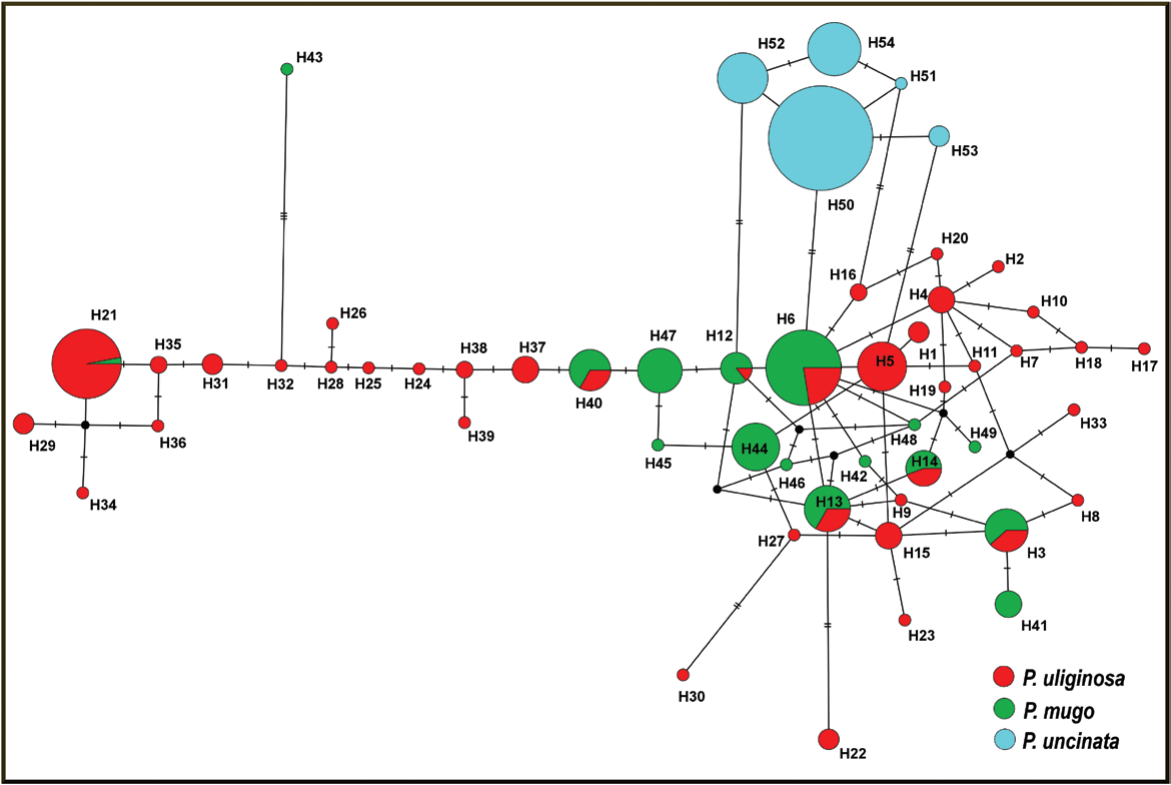
Median-joining network of haplotypes detected at 16 *mt*DNA regions in the taxa from the *Pinus mugo* complex. Sizes of the circles are proportional to haplotype frequencies, hatch marks represent numbers of nucleotide differences between them and shading indicates species.

Based on 16 polymorphic *mt*DNA markers we were able to identify 54 novel haplotypes in 363 trees from three pine species (Fig. 2; **see****Supporting Information—Table S2**). Overall, there was an abundance of minor frequency haplotypes with 37 haplotypes present in <1 % of all individuals (29 haplotypes were singletons and 8 were present only in 2–3 individuals). Particularly high number of singletons was found in *P. uliginosa*, especially in population UL_POL_Z (Zieleniec reserve), where an excess of rare haplotypes, with 13 singletons and highest value of haplotype diversity (*H*_d_ = 0.96), was observed (Table 1). Additionally, the highest number of haplotypes (*H*_n_ = 40) and average haplotype diversity (*H*_d_ = 0.91) were also detected in this species (Table 1). The average haplotype diversity was very similar for *P. mugo* (*H*_d_ = 0.87) but substantially lower for *P. uncinata* (*H*_d_ = 0.53). The three most common haplotypes were H50, H6 and H21 (Fig. 2). Haplotype H50 was exclusive to *P. uncinata* (except Spanish population from Valldelinares), H6 was almost fixed in *P. mugo* from Carnic Alps and occurred at low frequency in other dwarf mountain pine populations but was detected also in three peat bog pine populations (UL_POL_Z, UL_POL_W, UL_GER_MI) **[see****Supporting Information—Table S3**]. Haplotype H21 was dominant in *P. uliginosa* from Batorów reserve, but it was also present in three individuals in adjacent population from Zieleniec reserve and interestingly in one *P. mugo* individual from the Tatra Mts. Similar sharing of haplotypes between *P. mugo* from Polish mountains (both Tatra and Sudety Mts.) and *P. uliginosa* from Węgliniec reserve was found at haplotype H3. Except the mentioned shared common haplotypes between individuals in different populations (i.e. haplotypes H3, H6, H13), some local variants were also found to co-occur in neighbouring populations of different taxa (UL_GER_MI and M_AUT_K shared two haplotypes; UL_UKR_MS and M_UKR_MS shared one haplotype) **[see****Supporting Information—Table S3**, **Fig. S2****]**. The pattern of median-joining haplotype network revealed three main groups which coincide in general with three investigated species (Fig. 2), although haplotype sharing was found between *P. uliginosa* and *P. mugo*. Unique haplotypes were found only in *P. uncinata*.

Presence of strong and significant phylogeographic structure was inferred from considerable genetic differentiation among populations (*N*_ST_ > *G*_ST_; *P* < 0.001). Within species, population structure was observed in *P. uliginosa* and *P. mugo*, but not in *P. uncinata* (Table 2). After removing *P. uncinata* populations we still observed significantly greater *N*_ST_ than *G*_ST_ in the remaining populations based on PermutCpSSR analysis (data not shown).

**Table 2.** Genetic diversity estimates for *mt*DNA regions in *Pinus mugo* complex. *H*_T_—total gene diversity; *H*_S_—averaged gene diversity within populations; **significant at *P* = 0.01.

Species	*H*_T_	*H*_S_	*N*_ST_	*G*_ST_
*P. uliginosa*	0.98	0.72	0.605**	0.263
*P. mugo*	0.97	0.53	0.653**	0.457
*P. uncinata*	0.55	0.35	0.481	0.368
All	0.94	0.47	0.735**	0.505

**Figure 3.**
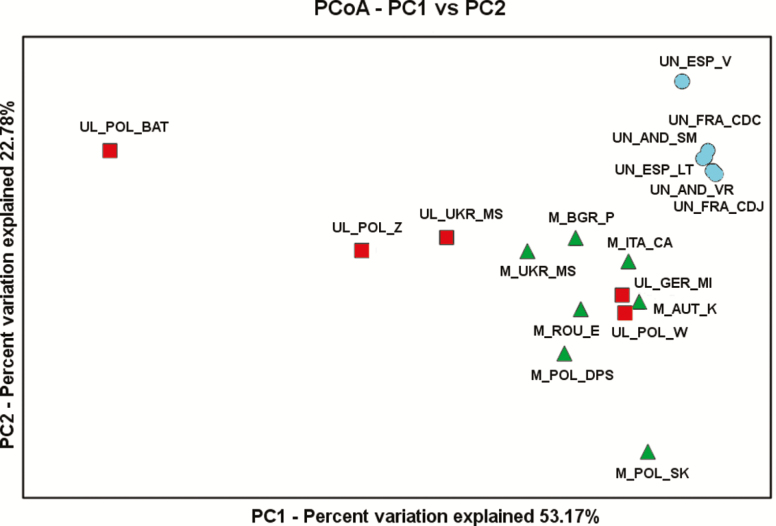
Results of PCoA based on average distances between studied populations calculated for a set of 16 *mt*DNA markers.

The evidence of population structure was further supported by results of the PCoA (Fig. 3). The majority of populations could be assigned to one of the three main clusters: (i) *P. mugo* together with *P. uliginosa* from Węgliniec and Mittenwald (UL_POL_W and UL_GER_MI); (ii) *P. uncinata* populations; (iii) *P. uliginosa*. However, two outlier populations including UL_POL_BAT and M_POL_SK showed distinct patterns of genetic variation and were isolated from other clusters. Similar relationships between the populations were observed in the UPGMA tree **[see****Supporting Information—Fig. S3****]**.

The result of SAMOVA at *K* = 2–17 is shown in **Supporting Information—Fig. S1**. The optimal number of groups, when the increment of Φ_CT_ was the largest, was four. The resulting SAMOVA groups did not exactly coincide with the taxa delineations but were similar to the pattern of genetic clusters indicated by the PCoA. The results show distinct character of *P. uncinata* populations (SAMOVA group II), similarity of two *P. uliginosa* and majority of *P. mugo* populations (SAMOVA group I), and unique character of the remaining *P. uliginosa* populations (SAMOVA groups III and IV) (Table 1). In the hierarchical AMOVA based on the division of populations into four groups, 60 % of the variation was due to differentiation between groups, while 24 % occured among populations within groups. Interestingly, the Mantel test showed statistically significant relationship between the genetic and geographic distances (*r* = 0.54, *P* < 0.001) suggesting presence of isolation by distance among populations. Nevertheless, when the three species were analysed separately, no statistically significant relationship was observed in any taxa (*P* > 0.05).

## Discussion

High-resolution molecular markers are needed for fine-scale population structure assessments and proper testing of phylogeograpic hypothesis. Difficulties involved in finding such variable markers, comparable in resolving power to animal *mt*DNA, have been severe in phylogeography of plants, especially non-model species with limited genomic resources (Beheregaray 2008). Due to slow mutation rate in plant mitochondrial genome, only two *mt*DNA markers including variation at *nad1* and *nad7* regions were developed for closely related pines from *P. mugo* complex. However, resolution of those markers was too low to provide any clear patterns of the species differentiation and populations structure. The application of more variable chloroplast DNA (*cp*DNA) markers, inherited in pines in paternal line and distributed at large geographical distances by pollen, was limited for closely related pine species (Palmer 1992; Wang and Wang 2014; Toth *et al.* 2017). In case of peat bog pine, which was grouped due to some similarities at biometric traits and incomplete reproductive isolation into larger taxonomic unit of the *P. mugo* complex (Christensen 1987), assessment of its genetic relationship at interspecific level based on *cp*DNA markers was especially hard. For instance, it was not possible to discriminate *P. uliginosa* from *P. mugo* and *P. uncinata* using variation of chloroplast DNA barcode regions (Celiński *et al.* 2017). Consequently, due to slow evolution of cytoplasmic genomes and very limited number of the regions screened for polymorphism, it was difficult to find species-specific genetic differences between those taxa and properly assess their intraspecific differentiation.

In advance to earlier studies our data provide some evidence of genetic variation within studied pine complex. Screening of a large set of newly developed mitochondrial markers together with previously known polymorphisms at two *mt*DNA regions delivered 54 novel haplotypes in 18, range-wide sampled, populations of the three investigated species. The results have substantially increased resolution of previous taxonomic investigations and population structure assessments in this pine species complex. Although there was extensive sharing of haplotypes between *P. mugo* and *P. uliginosa*, we were able to find fixed differences at two markers (*nad1* and PR13) that differentiate *P. uncinata* from other taxa in the complex. Low haplotype diversity and presence of species-specific haplotypes show clear genetic differentiation of *P. uncinata* supporting earlier suggestions of limited interspecific gene flow and its ongoing divergence (Wachowiak *et al.* 2013). The results are also in line with earlier karyotype studies of distinct heterochromatin patterns between *P. mugo* and *P. uncinata* (Bogunic *et al.* 2011). There are many factors that could have impact on the pattern of neutral genetic diversity including: level of gene flow, past climatic fluctuation, realized ecological niche and distribution range. The relatively low level of genetic diversity in *P. uncinata* is consistent with two general predictions: (i) lower levels of genetic diversity are expected for species with smaller distribution ranges; (ii) mountain populations tend to have lower haplotype diversity due to their peripheral location along an increasingly harsh elevation gradient (Herrera and Bazaga 2008). The results of chloroplast DNA variation in *P. uncinata* support those expectations (Dzialuk *et al.* 2017). Additionally, we did not find sharing of mitotypes between *P. uncinata* and *P uliginosa*, as the latter was generally more similar to *P. mugo*. This could be attributed to limited gene flow due to greater geographical distance between *P. uncinata* and *P. ulginosa* as compared to *P. mugo* and *P. uliginosa*. Contemporary ranges of *P. mugo* and *P. uncinata* are mostly disjunct but, some populations of the taxa overlap in Western Alps and could potentially form a hybrid zone. However, haplotype sharing through interspecific gene exchange seems unlikely taking into account the *cp*SSR results showing that the alpine *P. uncinata* population from Pyrenees forms a separate group as compared to the neighbouring *P. mugo* populations (Dzialuk *et al*. 2017). Our results clearly reject hypothesis about *P. uliginosa* being a marginal population of *P. uncinata* (Krzakowa *et al*. 1984), and they do not support suggestion that *P. uliginosa* may result from hybridization between *P. mugo* and *P. uncinata* (Dzialuk *et al.* 2017).

Our results provide clear evidence that *P. uliginosa* has surprisingly strong population structure with striking genetic differentiation among populations. The data indicate existence of different mitochondrial lineages in *P. uliginosa* and show that population from its *locus classicus* from Batorów reserve is the most diverged population within this taxon. Significant differentiation between populations distributed at relatively short geographical distance could be explained by limited gene flow and long-lasting separation of populations inhabiting disjunctive stands throughout their evolutionary history. Signs of differentiation were previously indicated based on some biometric features of cones and needles (e.g. Boratynska and Lewandowska 2009; Boratynska *et al.* 2015) and biochemical markers (e.g. Lewandowski *et al.* 2002; Wachowiak and Prus-Głowacki 2009). Nevertheless, it seems rather unlikely that such differentiation could result recently from pure isolation and genetic drift due to slow mutation rate of *mt*DNA in pines and late time of the formation of most European peatlands. Those areas started forming no earlier than at the last glacial maximum (LGM) and reached its peak around 9 ky ago (Gajewski *et al.* 2001). Possibly the remaining *P. uliginosa* stands represent populations of different origin that diverged long before the last glacial period and recolonized the current distribution from multiple sources. The existence of such cryptic central and north European refugia was postulated for other pines and forest tree species (Stewart and Lister 2001; Tzedakis *et al.* 2013; Ruiz-González *et al.* 2013).

High within-species divergence of *P. uliginosa* could also result from independent hybrid origin of different parental populations. Natural hybridization is recently recognized as an important process shaping evolution in many animal and plant species and it is well documented in conifers (Mallet 2005; Gao *et al.* 2012; Sun *et al.* 2014). Ecological divergence and adaptation to specific environmental niches facilitate spread of hybrids, despite co-occurrence with their parental types (Gross and Rieseberg 2005). The results of controlled crosses indicate incomplete reproductive isolation within the investigated pine complex and also with *P. sylvestris*, suggesting that hybridization between these taxa was highly possible in contact zones and could have contributed to *P. uliginosa* gene pool (Lewandowski *et al.* 2000; Wachowiak *et al.* 2005). Our data provide evidence on high genetic similarity between *P. uliginosa* and *P. mugo*. Differentiation in *P. uliginosa* could have arisen as a result of hybridization in postglacial secondary contact zones between populations of different ancestry representing these two species. Some of the shared haplotypes (i.e. haplotype H6) are widespread and common in both taxa, and thus may represent ancestral haplotypes acquired in distant past and retained in both lineages. We also detected less frequent haplotypes shared locally between neighbouring populations, for example H14 (UL_GER_MI and M_AUT_K) and H40 (UL_UKR_MS and M_UKR_MS). Considering weak reproductive barriers, hybridization in contact zones with mitochondrial capture between those two species seems possible. The observed pattern of haplotype distribution may thus reflect different influences of past (haplotypes shared in many populations and over large distance) and more recent (haplotypes shared locally) hybridization events on contemporary haplotype variation in *P. uliginosa*. However, we cannot exclude retention of ancestral polymorphism in those taxa and therefore nuclear markers would be needed to fully test this hypothesis.

Hybridization could also be invoked as the casual factor shaping unexpectedly high haplotype diversity found within *P. uliginosa* population from Zieleniec reserve. This population is particularly interesting as it represents a contact zone of three pine species (*P. uliginosa*, *P. mugo*, *P. sylvestris*) in a diverse habitat of the peat bog complex and it contains viable hybrid trees (Wachowiak *et al.* 2016). Although our sampling was restricted to trees classified based on morphological features as *P. uliginosa*, accidental inclusion of hybrid trees with *P. uliginosa*-like phenotype in our data set cannot be excluded. Presence of such exceptional number of haplotypes in individuals from Zieleniec reserve could result from acquisition of different mitotypes from the species involved in hybridization events. However, given the sheer number of haplotypes (18 in 27 individuals), this process alone can hardly explain mitochondrial variation observed in this population. Alternatively, *mt*DNA recombination mediated by hybridization events seems possible. Hypothesis of homologous recombination promoted by occasional parental leakage and heteroplasmy of *mt*DNA was previously proposed to explain high *mt*DNA variation in hybrid zone of spruce species (Jaramillo-Correa and Bousquet 2005) and this phenomenon was observed also in other conifers (Semerikov and Lascoux 2003; Semerikova and Semerikov 2014). Although paternal leakage of the mitochondrial genome has previously been reported to occur in other *Pinus* species (Wagner *et al.* 1991), there are no reports describing this phenomenon in species from *P. mugo* complex. Further tests with dense sampling of individuals from the contact zone of those three taxa and individuals from controlled crosses would be needed to support the hypothesis of exceptional haplotype diversity of *P. uliginosa* from Zieleniec reserve.

Our data provide evidence of high genetic variation and complex evolutionary history of the remnant *P. uliginosa* populations. Such a complex population structure, involving putative past and/or ongoing hybridization events, demands thoughtful consideration while developing conservation strategies for the taxa. Although not all endangered tree species are affected in the same manner by similar threats (Pautasso 2009), it seems evident that all *P. uliginosa* stands deserve preservation throughout the species range considering high genetic diversity and high degree of differentiation amongst populations. Extinction due to the decrease of the primary habitat is among the biggest threats to the peat bog pine. Active protection of all of these rare stands, coupled with creating conditions for its natural regeneration seems urgent. The existing genotypes should be protected by creating the clone archives (e.g. in form of cryopreserved somatic embryos) (Choudhury *et al.* 2014). To maintain diversity and reduce the threat of inbreeding in small populations, some level of human-mediated admixture between these geographically distinct populations should also be permitted allowing for some genetic rescue, an increase in effective population size and greater additive genetic variation. On the other hand, contemporary threat by genetic erosion in some populations (e.g. Zieleniec reserve) requires special attention, and invokes challenging questions, regarding conservation status of natural hybrids (Allendorf *et al.* 2001; Wachowiak *et al.* 2005; Stronen and Paquet 2013).

## Sources of Funding

The research was financially supported by the Polish National Science Centre (UMO-2015/19/B/NZ9/00024).

## Contributions by the Authors

B.Ł. and W.W. conceived the study; B.Ł. and J.Z. obtained and analysed genetic data; B.Ł. led the writing with support of W.W. and J.Z., who read and contributed to the final version of the manuscript.

## Conflict of Interest

None declared.

## Supporting Information

The following additional information is available in the online version of this article—

**Table S1.** Analysed loci and genotyping method.

**Table S2.** Major haplotypes and their frequency in the analysed taxa.

**Table S3.** Distribution of major haplotypes detected in studied pine taxa and populations.

**Figure S1.** Spatial analysis of molecular variance (SAMOVA).

**Figure S2.** Median-joining network of haplotypes detected at 16 mitochondrial DNA (*mt*DNA) regions in the taxa from the *Pinus mugo* complex.

**Figure S3.** Unweighted pair group method with arithmetic mean (UPGMA) phylogenetic tree of 18 studied pine populations.

Supplementary Material
