# Supplementary material for: Patterns of mtDNA variation reveal complex evolutionary history of relict and endangered peat bog pine (Pinus uliginosa)
Source: AoB Plants. 2019 Mar 14;11(2):plz015. doi: 10.1093/aobpla/plz015 (PMC6450798; doi:10.1093/aobpla/plz015)

Supplementary material

Supplementary Table S1. Analysed loci and genotyping method.

Marker name	Locus discovery	PCR primers (forward, reverse)	Product size	Position in amplicon, bp[a]	Restriction enzyme	Incubation temperature	Polymorphism	No. bands upon digestion
PR5	Donnelly et al. 2017: locus 3	ATTCCTGTGCTTGGTTGGGA<br>GGCGCTTACCCACACACTTA	570	279	DraI	37°	G / T	1 / 2
PR7	Donnelly et al. 2017: locus 5	TGAGTTCGTTGACCGCGTAA<br>TCAGGCGAGCTTGTGCTTTA	514	409	DraI	37°	A / C	2 / 1
PR13	Donnelly et al. 2017: locus 11	GATCGGGTCGGAGGCATAAT<br>AGTTGAAGCAAGCCAGCAAG	369	246	MseI	65°	C / A	2 / 1
PR14	Donnelly et al. 2017: locus 12	TTTACGAAGCCCTTGGCGAT<br>CTGAACCGGGTGTAGCCTTT	548	382	MseI	65°	G / T	1 / 2
PR15	Donnelly et al. 2017: locus 13	CATCCTCTCCTCTCGATGGC<br>GCTTTTGGCTTGGTGCGAAT	358	155	BpiI	37°	G / T	2 / 1
PR19	Donnelly et al. 2017: locus 17	CGGAGCGAGGTGAAGAAACT<br>GCGAGAAGCAGTAGTGGGTT	593	413	DraI	37°	G / T	1 / 2
PR20	Donnelly - not publ.	GTTCCTACGATCCAGCCAGG<br>ACCATGGATTCTTCGGACGG	382	173	MvaI	37°	C / A	3 / 2
PR21	Donnelly et al. 2017: locus 18	TCCGATGATGAGGTGGAGGT<br>AGTTGAAGGCAGGAAGGTCG	522	421	HincII	37°	G / T	2 / 1
PR24	Donnelly et al. 2017: locus 19	TGCATTCTGGCTGGCTTTCT<br>GGCGTCGATAGACTCGGTTT	434	272	BshNI	37°	T / G	1 / 2
PR25	Donnelly et al. 2017: locus 20	GGCATGTCCGCTATGGAAGT<br>AGGCTCCGGAAGTACCTGT	398	123	BtsCI	55°	G / T	1 / 2
PR29	Donnelly et al. 2017: locus 22	GGTTGGTTGATCCATCCGGT<br>CCGGCTTGGGTACGTCTTTT	558	226	BtsCI	55°	G [b]	1 / 2
PR30	Donnelly - not publ.	ACTTACATTGACCGGCGGAT<br>CACACATCTAGGGCACAGGG	301	194	Alw26I	37°	G / T	1/ 2
PR31	Donnelly et al. 2017: locus 23	TGCGACCTGTGAATGGATGT<br>CGGCGGTTCTAGCCTTGATT	558	293	VspI	37°	G / T	1 / 2
PR32	Donnelly - not publ.	ACCCTCCTTCAACTGATGCG<br>CCTCAACCAACCGTCAGTCA	407	127	PfeI	37°	G / T	1 / 2
PR34	Donnelly - not publ.	GAACCCCCTCTTGCCTTGAT<br>TTCGTGACGGTCCAATTCCA	398	176 -184	na	na	IN/DEL	na

[a] primers excluded, [b] monomorphic in this data set

Supplementary Table 2. Major haplotypes and their frequency in the analysed taxa.

Marker	PR5	PR7	PR13	PR14	PR15	PR19	PR20	PR21	PR24	PR25	PR30	PR31	PR32	PR34*	*nad1**	*nad7**	Frequency [% (N of samples)] ALL	PUG	PM	PUN
**H3**	G	A	C	G	G	T	C	G	T	G	G	G	G	IN	a	A	3.6 (13)	3.8 (5)	7.2 (8)	0.0 (0)
**H4**	T	C	C	G	G	G	C	G	T	G	G	G	G	DEL	a	A	1.4 (5)	3.8 (5)	0.0 (0)	0.0 (0)
**H5**	G	C	C	G	G	T	C	G	T	G	G	G	G	DEL	a	A	4.7 (17)	12.8 (17)	0.0 (0)	0.0 (0)
**H6**	G	C	C	G	G	G	C	G	T	G	G	G	G	DEL	a	A	11.0 (40)	6.8 (9)	27.9 (31)	0.0 (0)
**H12**	G	C	C	T	G	G	C	G	T	G	G	G	G	DEL	a	A	1.9 (7)	0.8 (1)	5.4 (6)	0.0 (0)
**H13**	G	A	C	G	G	G	C	G	T	G	G	G	G	DEL	a	A	4.1 (15)	3.8 (5)	9.0 (10)	0.0 (0)
**H14**	G	A	C	G	G	G	C	G	G	G	G	G	G	DEL	a	A	2.8 (10)	3.0 (4)	5.4 (6)	0.0 (0)
**H15**	G	A	C	G	G	T	C	G	T	G	G	G	G	DEL	a	A	1.4 (5)	3.8 (5)	0.0 (0)	0.0 (0)
**H21**	T	C	C	T	T	G	A	T	T	T	T	G	G	DEL	a	B	9.4 (34)	24.8 (33)	0.9 (1)	0.0 (0)
**H37**	G	C	C	T	G	G	C	G	T	T	G	G	T	DEL	a	B	1.4 (5)	3.8 (5)	0.0 (0)	0.0 (0)
**H40**	G	C	C	T	G	G	C	G	T	T	G	G	T	DEL	a	A	3.3 (12)	3.0 (4)	7.2 (8)	0.0 (0)
**H41**	G	A	C	G	G	T	C	G	T	G	G	G	T	IN	a	A	1.4 (5)	0.0 (0)	4.5 (5)	0.0 (0)
**H44**	G	C	C	G	G	T	C	G	T	T	G	G	G	DEL	a	A	4.4 (16)	0.0 (0)	14.4 (16)	0.0 (0)
**H47**	G	C	C	T	G	G	C	G	T	T	G	G	G	DEL	a	A	3.9 (14)	0.0 (0)	12.6 (14)	0.0 (0)
**H50**	G	C	A	G	G	G	C	G	T	G	G	G	G	DEL	b	A	21.2 (77)	0.0 (0)	0.0 (0)	64.7 (77)
**H52**	G	C	A	T	G	G	C	G	T	G	G	G	G	DEL	b	A	5.0 (18)	0.0 (0)	0.0 (0)	15.1 (18)
**H54**	G	C	A	T	G	G	C	G	T	G	G	T	G	DEL	b	A	5.5 (20)	0.0 (0)	0.0 (0)	16.8 (20)
																	**86.2 (313)**	**69.9 (93)**	**94.6 (105)**	**96.6 115)**
																**Minor haplotype number**	**37**	**28**	**6**	**3**
																**Minor haplotype frequency**	**13.8**	**30.1**	**5.4**	**3.4**

* PR34: IN – TCATCAATC, DEL– --A—AAGA*; nad1*: variants as in Naydenov et al. 2007; *nad7*: variants as in Soranzo et al. 2000;

Table S3. Distribution of major haplotypes (present in at least 3 individuals) detected in studied pines taxa and populations.

**Haplotype** / **Population**	H3	H4	H5	H6	H12	H13	H14	H15	H21	H37	H40	H41	H44	H47	H50	H52
UL_POL_W	x	x	x	x	x	x										
UL_GER_MI			x	x		x	x	x								
UL_POL_BAT									x							
UL_POL_Z				x		x			x							
UL_UKR_MS										x	x					
M_POL_SK	x											x				
M_POL_DPS	x				x				x							
M_AUT_K				x	x	x	x	x								
M_UKR_MS											x					
M_ROU_E					x								x			
M_BGR_P				x										x		
M_ITA_CA				x	x											
UN_AND_VR															x	x
UN_AND_SM															x	
UN_ESP_LT															x	x
UN_ESP_V																x
UN_FRA_CDJ															x	
UN_FRA_CDC															x	x
P. uliginosa	x	x	x	x	x	x	x	x	x	x	x					
P.mugo	x			x	x	x	x	x	x		x	x	x	x		
P. uncinata															x	x

Supplementary Figure S1. Spatial analysis of molecular variance (SAMOVA). Results of the spatial analysis of molecular variance showing the genetic affinity between 18 populations of three different pine species. The most likely subdivisions of the whole distribution area consisted of four groups, when the increment of F_{CT} was the largest ($\Delta\Phi_{CT}$ = 0.04).

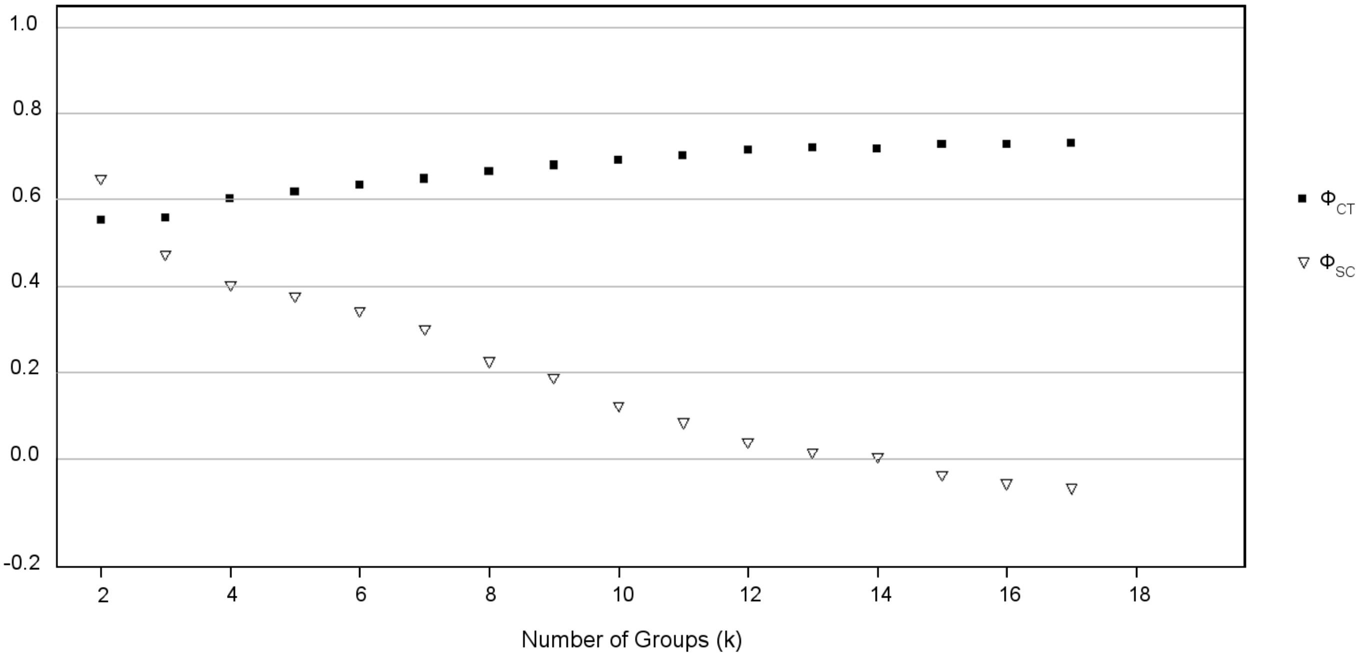

Supplementary Figure S2. Median-joining network of haplotypes detected at 16 *mt*DNA regions in the taxa from the *P. mugo* complex. Size of the circles are proportional to haplotype frequencies, hatch marks represent numbers of nucleotide differences between them and shading indicates specific populations.

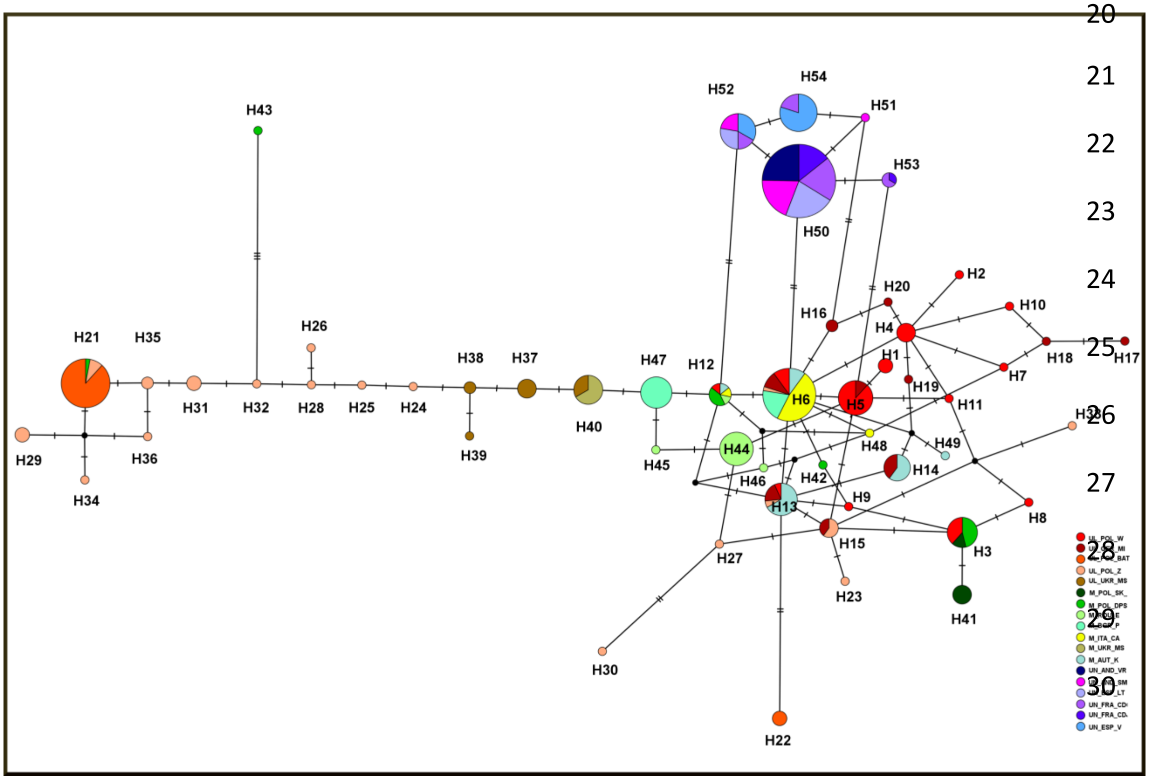

Supplementary Figure S3. UPGMA phylogenetic tree of 18 studied pine populations. The tree is constructed using genetic distances based on number of differences between all polymorphic *mt*DNA sites. The tree is drawn to scale with the branch lengths representing the evolutionary distances, the scale can be seen at the bottom of the tree.

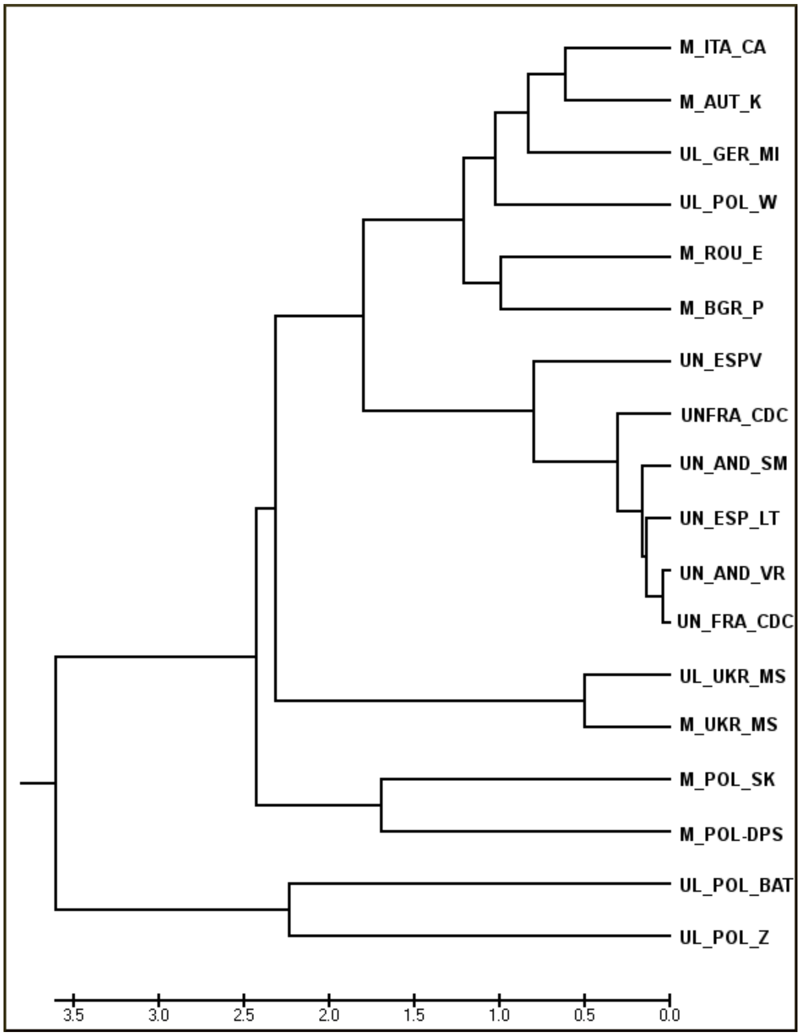

Supplement: Supplementary Material [file plz015_suppl_supplementary_material.pdf]
